# Polypoidal Choroidal Vasculopathy Complicating Retinal Laser in Quiescent Uveitis

**DOI:** 10.1155/2019/6147063

**Published:** 2019-01-17

**Authors:** Tomas R. Burke, Susan L. Lightman

**Affiliations:** ^1^Moorfields Eye Hospital, London, UK; ^2^University College London Institute of Ophthalmology, London, UK

## Abstract

A 58-year-old Afro-Caribbean gentleman with a diagnosis of quiescent systemic lupus erythematosus- (SLE-) related occlusive retinal vasculitis was previously treated with sector pan-retinal photocoagulation in his right eye to control temporal retinal neovascularization. At routine review he was found to have a focal area of subretinal fluid in the temporal macula sparing an ischaemic fovea. Fundus fluorescein angiography and indocyanine green angiography confirmed a branching vascular network (BVN) and terminal polys (i.e., polypoidal choroidal vasculopathy (PCV)). Interestingly, the BVN arose within an old laser scar. To our knowledge this is the first report of PCV in uveitis in an Afro-Caribbean patient and of the lesions arising within a laser scar.

## 1. Introduction

Choroidal neovascularization (CNVM) is a well-recognised complication of inflammatory ocular diseases, particularly those that affect the posterior segment [1]. CNVM are also known to arise secondary to iatrogenic causes such as previous laser therapy [2]. It has been proposed that a discontinuity in the Bruch's membrane-RPE complex is the initiating event in the development of CNVM [3, 4]. Reviewing the literature reveals that there was 1 previous report of of polypoidal choroidal vasculopathy (PCV) in the context of SLE-related uveitis [5], and that patient was of Chinese (i.e. Asian) origin. We present a case of PCV developing in an Afro-Caribbean patient with marked macular ischaemia secondary to SLE-related occlusive retinal vasculitis who has previously been treated with sector pan-retinal photocoagulation (PRP) laser.

## 2. Case Report

A 58-year-old Afro-Caribbean gentleman attended routine follow-up with a long-standing history of reduced vision (right > left eye), but with no acute visual symptoms. His past ocular history included quiescent bilateral occlusive retinal vasculitis secondary to SLE, with neovascularization in the right temporal retina secondary to a branch retinal vein occlusion that required sector pan-retinal photocoagulation laser 6 years previously. His SLE was also associated with nephritis and was controlled with Mycophenolate Mofetil 1.5g BD.

**Figure 1:**
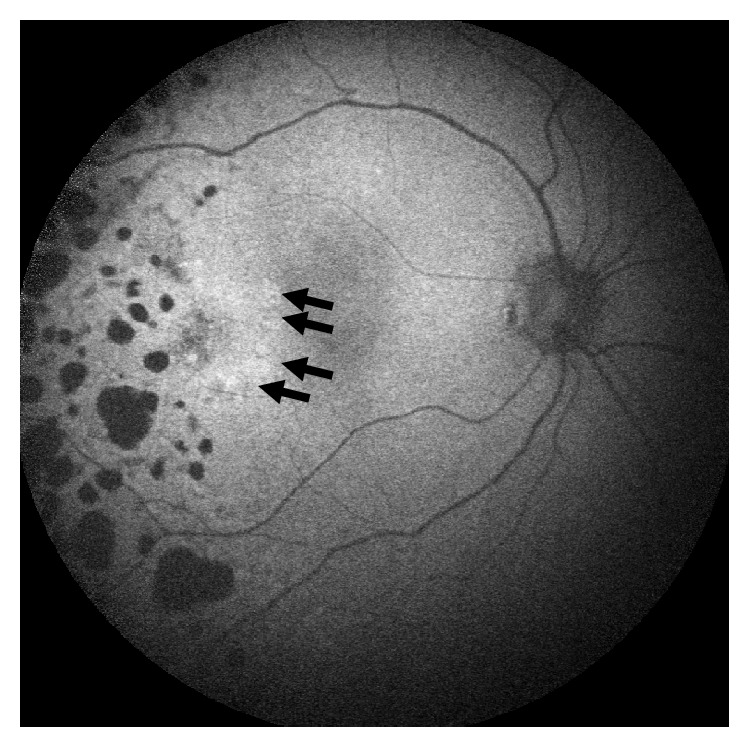
Fundus autofluorescence (FAF) right eye at baseline. Note the laser scars appears as heterogeneous hypoAF foci in the temporal fundus of the right eye. Nasal to these, in the temporal macula, note the curvilinear group of faint hyperAF foci (black arrows).

**Figure 2:**
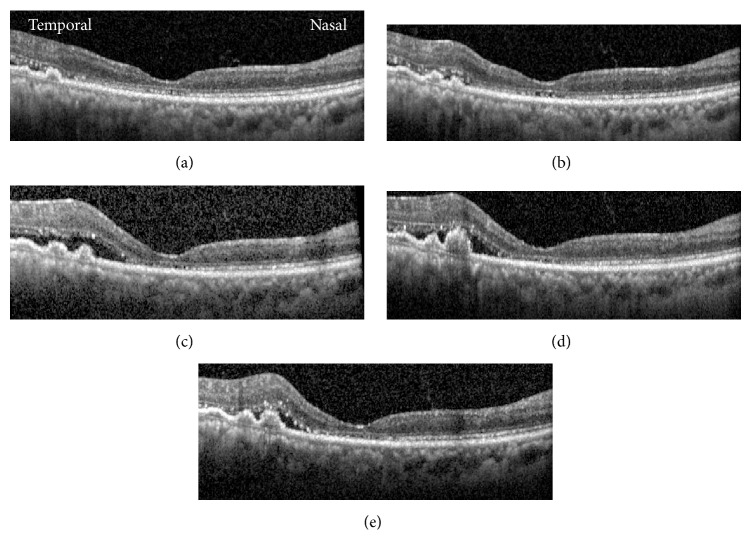
Spectral domain-OCT images of right macula from baseline (a) through follow-up at various intervals: +4 months (b), +7 months (c), +8 months (d), and +9 months (e). Note the waxing and waning of subretinal fluid, the variations in the appearance of the temporal pigment epithelial detachments, and the fovea remaining dry throughout follow-up.

**Figure 3:**
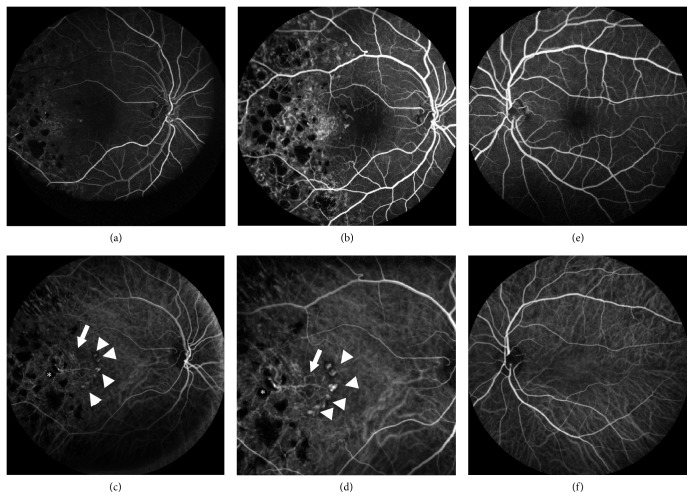
Fundus fluorescein angiogram (55°) of right eye (a, b) and left eye (e). Indocyanine green angiography (ICGA) of right eye (55° c, 30° d) and left eye (f). The branching vascular network (BVN, white arrows) and terminal hyperfluorescent polyps (white arrowheads) were clearly visible on ICGA. These also demonstrated a characteristic hypofluorescent halo. Note that the BVN originates from a hypofluorescent laser scar (*∗* in c and d). FFA of the right eye revealed macular ischaemia. FFA and ICGA of the left eye were normal.

**Figure 4:**
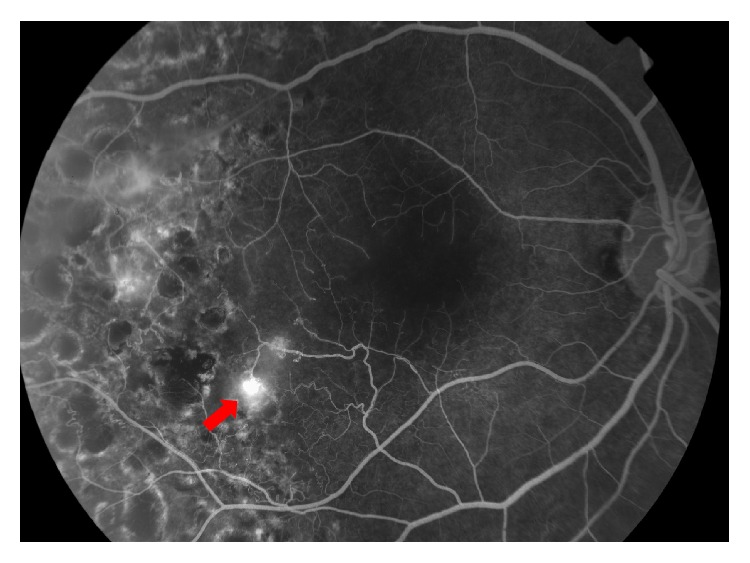
Fundus fluorescein angiogram of the right eye carried out 4 years before development of polypoidal lesions. Macular ischaemia is evident, together with active retinal neovascularization in the infero-temporal macula (red arrow). Previous laser retinal scars are also visible temporally.

**Figure 5:**
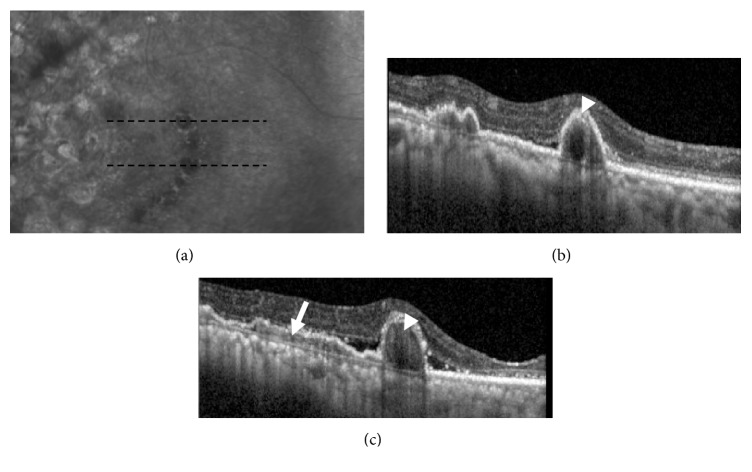
Infra-red image (a) of right eye with corresponding enhanced depth-imaging (EDI) spectral domain-OCT of right macula at the 9 months follow-up time-point. The black dashed line indicates to relevant position of the EDI images (upper b, lower c). Note the “double-layer” sign in image (c) (white arrow) as a hyper-reflective area in the space between the retinal pigment epithelium and Bruch's membrane. This suggests the location of the branching vascular network. Within the pigment epithelial detachments, hyper-reflective lesions can be seen that most likely represent polyps (white arrow-head in b, c).

**Figure 6:**
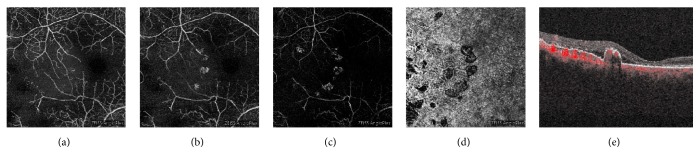
OCT-Angiography (OCT-A) images of the right eye at the 7-month follow-up time-point. The superficial (a) and deep (b) OCT-A slabs revealed macular ischaemia. The polyps were visible in (b), the avascular (c) and choriocapillaris (d) slabs. Flow within the polyps was seen on the cross-sectional OCT-A image (e).

Best-corrected visual acuity was stable (right eye 6/36, left eye 6/18). No intraocular inflammation was observed. Intraocular pressures were normal. No active neovascularization was observed, though a frond of partially ibrosed neovascularization was present supero-temporally in the right fundus. His maculae were featureless but for a group of faint pinkish lesions in the right temporal macula. Fundus autofluorescence (FAF, Figure 1) and spectral-domain optical coherence tomography (SD-OCT, Figure 2) were carried out using the Heidelberg Spectralis (Heidelberg Engineering, Heidelberg, Germany). FAF revealed laser scars as temporal hypoAF areas and there was a curvilinear group of hyperAF lesions nasal to these. SD-OCT revealed a thinned dry fovea with temporal macular subretinal fluid (SRF) observed over a group of pigment epithelial detachments (PED). As the patient was asymptomatic, observation was initiated and a 4-month follow-up arranged. At review, repeat imaging revealed progression, though the fovea remained dry (Figure 2). Fundus fluorescein angiography (FFA) and indocyanine green angiography (ICGA) (Heidelberg Spectralis, Heidelberg Engineering, German) revealed a branching vascular network (BVN), arising within a laser scar, with terminal hyperfluorescent polyps (i.e. PCV, Figure 3). Dilated ‘pachyvessels' were obvious in the right eye of this patient on ICGA, including areas away from the focus of the BVN. The fellow eye had a normal ICGA without pachyvessels. FFA (Figure 4, Topcon Corp, Tokyo, Japan) performed 4 years previously revealed macular ischemia, active retinal neovascularization and previous laser therapy, however there was no evidence of PCV on that angiogram. Over the course of follow-up (9 months), the subfoveal space and vision were not affected (Figure 2). Enhanced-depth imaging OCT revealed with the ‘double-layer' sign and polyps within the temporal macular PEDs (Figure 5). OCT-Angiography images (Zeiss Angioplex) demonstrated widespread macular ischaemia in the right macula, together with the BVN and polyps (Figure 6).

## 3. Discussion

The term PCV was introduced in 1982 (Yannuzzi LA. Idiopathic Polypoidal Choroidal Vasculopathy. Presented at the Macula Society Meeting, Miami, Florida) and published in 1990 to describe a choroidal vasculopathy leading to serous and haemorrhagic pigment epithelial detachments [6]. It is more prevalent in Asian patients, but otherwise there is limited high quality epidemiological data due to the difficulty in confirming the diagnosis [7]. These lesions consist of a BVN with polypoidal lesion at its edge, and appear under the retinal pigment epithelium. ICGA remains the gold-standard for PCV diagnosis, where the typical finding is that of polypoidal lesions which hyperfluoresce over the run of the angiogram with or without the presence of a BVN. Previously it has been suggested that vascular polyps represent a Type 1 neovascular process [8]. Polypoidal lesions have been reported in a range of conditions [9–13], including those that fall within the pachychoroid spectrum [14–16]. One previous report describes the development of PCV in a Chinese patient with SLE. Choroidal vasculitis was proposed as a mechanism for PCV in SLE incited by common complement pathway activation. Importantly, it has previously been reported that systemic inflammatory markers including C-reactive protein and certain cytokines have been elevated locally and/or systemically in patients with PCV [17, 18]. In our patient, the ICGA highlighted dilated pachyvessels in the right eye away, including regions away from the BVN and laser. This suggests a diffuse pathology of the choroid in the right eye. It is interesting, therefore, that the BVN lesions appeared to arise within a previous laser scar. However, it is not possible to state definitively if the uveitis history+/-laser was relevant for PCV development, particularly as the ocular inflammation had been quiescent for years. Nonetheless this case appears to be the first report of PCV arising within the region of a previous laser scar in the context of uveitis. Debate continues in the literature regarding the exact mechanism of development and even location of the abnormal vessels in PCV [7].

As PCV is more difficult to diagnose than CNVM (often requiring ICGA) and given the divergent management requirements, we must consider that this phenomenon may be under-reported in the iatrogenic- and inflammatory-CNVM literature and maintaining a high index of suspicion for secondary PCV may be justified.

## Data Availability

The clinical data used to support the findings of this study are included within the article.
